# LINC01214 Promotes Non-Small Cell Lung Cancer Through the miR-497-3p/HSP90AB1 Axis

**DOI:** 10.1155/carj/5575392

**Published:** 2025-10-30

**Authors:** Guangfu Xu, Ling Zhang, Fei Li, Wenwen Han, Hailong Sun, Jiangtao Cao

**Affiliations:** ^1^Department of Pathology, Zhejiang Provincial People's Hospital, Hangzhou 310014, China; ^2^Department of Pulmonary and Critical Care Medicine, The People's Hospital of Dadukou District Chongqing, Chongqing 400084, China; ^3^Department of Medical Imaging, Dongying People's Hospital, Dongying 257091, China; ^4^Department of Respiratory and Critical Care Medicine, First Medical Center of Chinese PLA General Hospital, Beijing 100853, China

**Keywords:** HSP90AB1, lncRNA LINC01214, miR-497-3p, NSCLC

## Abstract

**Objective:**

This study aimed to explore the potential of LINC01214 in providing prognostic and therapeutic insights, thereby offering valuable references for the research of non-small cell lung cancer (NSCLC).

**Methods:**

122 NSCLC subjects were recruited. The molecular level was quantified by qPCR and WB. Kaplan–Meier estimated the prognostic effects and Cox regression analyses the hazard ratio. The cell activities including proliferation, apoptosis, and migration/invasion were evaluated by CCK-8, flow cytometry, and Transwell. The regulatory axis of LINC01214/miR-497-3p/HSP90AB1 was verified by the dual luciferase reporter assay and rescue experiments.

**Results:**

LINC01214 increased both in the malignant tissues and cell lines of NSCLC. Mortality was increased in NSCLC patients with high LINC01214 levels. LINC01214 was an independent risk predictor of prognosis in NSCLC. Silencing of LINC01214 inhibited the NSCLC cell proliferation, migration, and invasion and promoted apoptosis. The abundance of miR-497-3p showed an opposite trend to LINC01214. LINC01214 could target miR-497-3p and negatively correlate with miR-497-3p. The inhibitory effect of LINC01214 on cell activity was reversed by miR-497-3p. HSP90AB1 was predicted and further confirmed as the target of miR-497-3p. The LINC01214/miR-497-3p/HSP90AB1 axis regulated NSCLC cell proliferation, migration, and invasion.

**Conclusion:**

LINC01214, a potential biomarker, contributed to the progression of NSCLC through the miR-497-3p/HSP90AB1 axis by promoting cell proliferation and motility.

## 1. Introduction

Lung cancer, a malignant tumor with the highest morbidity and mortality [1], is predominantly comprised of non-small cell lung cancer (NSCLC), which accounts for over 80% of cases [2]. Because early symptoms are nonspecific and there is no effective diagnostic test, most cases of NSCLC are often in the middle and advanced stages when diagnosed [3, 4]. Based on the patient's condition, the most common clinical treatments include surgery, radiotherapy, chemotherapy, and targeted therapy [5]. Despite the use of multiple therapeutic options, the prognosis of NSCLC patients remains poor [6]. Therefore, the treatment of NSCLC has become a difficult point in clinical treatment. Exploring new biomarkers holds significant clinical importance for enhancing the treatment outcomes of patients.

Long noncoding RNAs (lncRNAs), characterized by sequences longer than 200 nucleotides, do not encode proteins [7] but are engaged in various physiological processes such as tumorigenesis, cell cycle regulation, differentiation, and apoptosis [8]. Furthermore, relevant studies indicated that lncRNAs are essential in the diagnosis, prognosis, and drug resistance in targeted therapies for malignant disease [9]. For example, lncRNA NORAD may bind to miR-520a-3p, affecting the PI3k/Akt/mTOR pathway to accelerate NSCLC progression [10]. Downregulated LINC-PINT is associated with adverse prognosis and advanced stage in NSCLC [11]. Previous studies showed that LINC01214 is elevated in NSCLC, but its mechanism is unclear [12]. Actually, we found three articles discussing LINC01214's expression in different diseases, one suggesting it could be a diagnostic marker for psoriasis [12–14]. While its expression in lung cancer has been studied, its functional role and molecular interactions remain unknown, indicating a need for further research to understand its potential mechanism. LncRNAs play a critical role mainly by acting as competing RNAs of microRNAs (miRNAs) [15]. Potential downstream miRNAs of LINC01214 were identified using the lncRNASNP database, including miR-497-3p, which has recently been recognized for its tumor-relative role in various cancers. For example, lncRNA ELFN1-AS1 plays a role in ovarian cancer through direct interaction with miR-497-3p [16]. Thyroid cancer progression is delayed by inhibiting PAK1/β-catenin pathway elevation through miR-497-3p [17]. miR-497-3p is abnormally expressed and regulates the IGF-1R pathway in lung cancer, making it a promising target for addressing drug resistance [18]. According to the above review, LINC01214 and miR-497-3p are differentially expressed in NSCLC and may serve as targets for combined interventions. However, the mechanism of LINC01214 is not yet fully understood, and the regulatory relationship between LINC01214 and miR-497-3p in the advancement of NSCLC still requires validation.

The present study aims to explore the potential impact of LINC01214 and its regulatory axis in NSCLC by examining the expression and function of the LINC01214/miR-497-3p axis in clinical samples and various cell lines. This research hopes to provide valuable insights for diagnosis and prognosis, as well as a deeper understanding of the molecular mechanisms underlying NSCLC.

## 2. Materials and Methods

### 2.1. Ethical Statement

Subjects were enrolled with an informed consent form signed before enrollment. The study protocol was reviewed and approved by the Ethics Committee of Dongying People's Hospital (Ethics Approval No.: 20161002), with approval granted for the period from October 2016 to October 2019. The study was conducted in accordance with the ethical standards of the institutional research committee and the principles of the Declaration of Helsinki.

### 2.2. Study Subjects

A total of 122 NSCLC patients who underwent tumor resection in Dongying People's Hospital from January 2017 to May 2019 were enrolled. There was no preoperative neoadjuvant therapy. All patients were diagnosed with NSCLC according to the 5th edition of the WHO lung tumor classification guidelines. Exclusion criteria [1] were the following: patients with nonprimary lung cancer such as metastatic cancer [2], complicated with other active malignant tumors [3], patients with incomplete clinical data or follow-up records [4], and patients who received antitumor therapy before surgery.

### 2.3. Prognostic Follow-Up

All patients were followed up. Follow-up began after treatment and was completed until the occurrence of an endpoint event or 5 years after treatment. The endpoint event was death or recurrence, and the follow-up results and time of occurrence were recorded.

### 2.4. Clinical Samples Collection

Tumor and adjacent normal tissue (3 cm from the edge of tumor tissue) were collected during surgery. The collected tumor tissue and adjacent normal tissue were quickly stored in liquid nitrogen.

### 2.5. RT-qPCR

Trizol reagent was used to extract total RNA. RNA concentrations and quality were measured, and RNAs with OD260/OD280 results close to 2.0 were reverse transcribed into cDNA. The PCR reaction system was configured according to the instructions of the SYBR Premix Ex Taq II kit (Takara, Japan). The relative expressions of LINC01214, miR-497-3p, and HSP90AB1 were detected by fluorescence quantitative PCR and quantified by the 2^−ΔΔCt^ method. GAPDH and U6 were used as internal controls.

### 2.6. Western Blot

Total protein was extracted from NSCLC cells using RIPA lysis buffer supplemented with protease and phosphatase inhibitors (Beyotime, China). The protein concentration was determined using a BCA Protein Assay Kit (Beyotime). Equal amounts of protein were separated on 7.5% SDS-PAGE gels (Epizyme Biotech, China) and transferred onto PVDF membranes (Millipore, USA). After incubation with WB blocking solution (Beyotime), the membranes were incubated overnight at 4°C with primary antibodies against HSP90AB1 (1:1000, CST, USA) and GAPDH (1:5000, Proteintech, China) as a loading control. After washing, the membranes were incubated with HRP-conjugated secondary antibodies (1:5000, Proteintech) for 1 h at room temperature. Protein bands were visualized using an enhanced chemiluminescence (ECL) kit (Beyotime) and quantified using ImageJ software.

### 2.7. Cell Culture and Cell Transfection

NSCLC cell lines (H-2009, PC-9, A549, and H1299) and normal cell lines (HBE) were purchased from the Cell Bank (Shanghai). Cell lines were cultured in MEM complete medium (added with 10% FBS). The medium was incubated at 37°C with 5% CO_2_. The frequency of subcultures is approximately every 3 days (at a ratio of 1:2). Subsequent experiments were performed when the cells were 80% confluent. Vectors were purchased from Bioengineering (Shanghai). pcDNA3.1 was used for the empty vector. The transfection vectors included small interfering RNA of LINC01214 (si-LINC01214) and a negative control (si-NC). The target sequence of si-LINC01214 was GAACCCACAUCUACCAUGA. Mimics and inhibitors of miR-497-3p were provided by RiboBio (Guangzhou). Lipofectamine 2000 (Thermo, USA) was used to transfect NSCLC cell lines. Two NSCLC cell lines (PC-9, A549) with the highest expression of LINC01214 were selected for subsequent experiments. To investigate the regulatory axis LINC01214/miR-497-3p/HSP90AB1, rescue experiments were performed in PC-9 cells. Cells were divided into four groups: control, miR-497-3p mimic, miR-497-3p mimic + empty vector (pcDNA3.1), and miR-497-3p mimic + pcDNA3.1-HSP90AB1. Transfections were carried out using Lipofectamine 2000 (Thermo) according to the manufacturer's protocol.

### 2.8. Dual Luciferase Report

The online database lncRNASNP predicts the binding sites of LINC01214 and miR-497-3p. Wild-type (WT) and mutant (MUT) LINC01214 sequences containing miR-497-3p binding sites were ligated to the pmirGLO dual luciferase reporter vector to obtain recombinant plasmids WT-LINC01214 and MUT-LINC01214. The plasmids were transfected with mimics or inhibitors of miR-497-3p into NSCLC cell lines (PC-9, A549) using Lipofectamine 2000 (Thermo, USA). There were three compound holes in each group. The medium was replaced 6 h after transfection, and the culture was continued. After 48 h, the medium was aspirated and assayed according to the manufacturer's instructions (Promega, USA). The relative fluorescence activity was calculated. A dual luciferase assay for HSP90AB1 and miR-497-3p was performed as above.

### 2.9. CCK-8 and Transwell Assay

Cell proliferation was measured by CCK-8 using 96-well plates (in triplicate per group). 10 μL of the CCK-8 solution was added to each well at different culture time points (0, 24, 48, and 72 h) and incubated for 2 h. Absorbance value was detected at 450 nm using a microplate reader.

Cell invasion and migration were assayed by Transwell and observed by light microscopy and counted. For invasion assays, Matrigel gel was diluted 1:8 in PBS buffer, evenly added to the upper chamber surface of the bottom membrane of the chamber, and incubated for 3 h to polymerize the Matrigel into a film to mimic the vascular basement membrane. After gelation, cells were seeded. For migration assays, the upper and bottom chambers of the Transwell 24-well plate were seeded with NSCLC cells (PC-9, A549, 1 × 10^5^ cells/well) and medium containing 10% FBS, respectively. After 24 h, the upper cells were gently wiped off, and the bottom cells were stained with 0.2% crystal violet.

### 2.10. Apoptosis Assay

Apoptosis was evaluated using the Annexin V-FITC/PI Apoptosis Detection Kit (Beyotime) according to the manufacturer's protocol. After transfection, NSCLC cells were harvested, washed twice with cold PBS, and resuspended. Cells were then stained with Annexin V-FITC and propidium iodide (PI) for 15 min in the dark at room temperature. The samples were analyzed by flow cytometry (BD FACSCalibur, USA) within 1 hour.

### 2.11. Data Analysis

The data were analyzed using SPSS 23.0 and GraphPad Prism 9.0. Significance was calculated using an independent *t*-test or two-way ANOVA. Count data were expressed as cases and evaluated by the chi-square test. Kaplan–Meier and Cox regression assayed the survival curve and independent prognostic factors, respectively. The correlation coefficient was calculated using the Pearson analysis. Statistical power analysis was performed using the power.*t*-test function in R.

## 3. Results

### 3.1. Clinical Characteristics of LINC01214 in NSCLC

**Figure 1:**
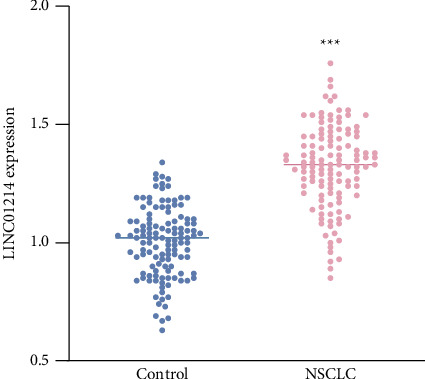
LINC01214 was significantly elevated in the tumor tissues of NSCLC patients. ^∗∗∗^*p* < 0.001.

The abundance of LINC01214 increased by approximately 40% in NSCLC tissues (*p* < 0.001, Figure 1), and its upregulation trend was consistent with the TCGA cohort (Supporting Figures available here). According to the mean expression level in NSCLC, two groups were obtained named high/low LINC01214. It was observed in Table 1 that the high level of LINC01214 was closely related to the indexes including differentiation (*p*=0.039), lymph node metastasis (*p*=0.023), and TNM stage (*p*=0.043).

**Figure 2:**
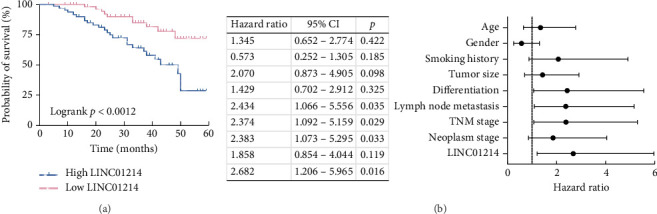
Prognostic value of LINC01214 in NSCLC. (a) Lower LINC01214 expression was favorable to the prognosis in the Kaplan–Meier analysis. (b) LINC01214 was an independent risk factor in the Cox analysis.

The Kaplan–Meier curve demonstrated that the survival rate was higher in the low LINC01214 group (Figure 2(a)). Age 60 was selected as the stratification threshold [19], but Cox regression analysis indicated that age was not an independent prognostic factor for NSCLC (Figure 2(b)). Cox regression analysis revealed that LINC01214 (HR: 2.682, 95% CI: 1.206–5.965, *p*=0.016) and three tumor grade indicators, differentiation (HR: 2.434, 95% CI: 1.066–5.556, *p*=0.035), lymph node metastasis (HR: 2.374, 95% CI: 1.092–5.159, *p*=0.029), and TNM stage (HR: 2.383, 95% CI: 1.073–5.295, *p*=0.033), were independent risk predictors for NSCLC prognosis (Figure 2(b)).

### 3.2. LINC01214 Binds to miR-497-3p Targeting

**Figure 3:**
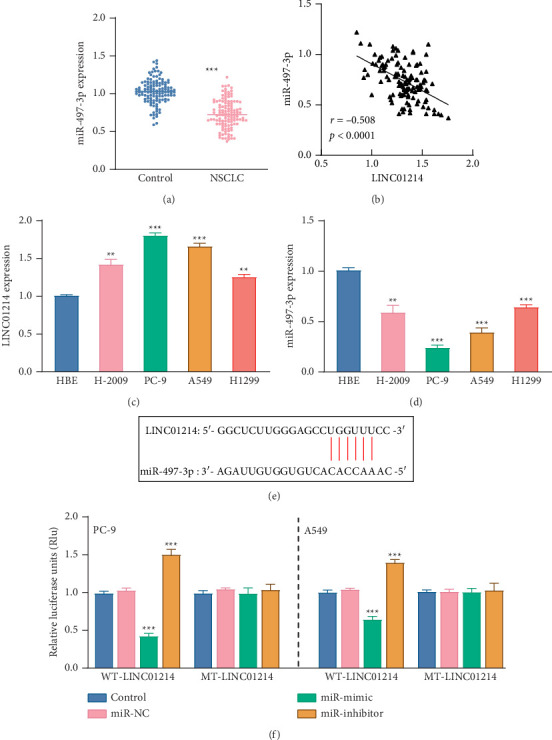
The relationship between LINC01214 and miR-497-3p. (a) miR-497-3p was significantly decreased in the NSCLC tissues. (b) Negative correlation between the abundance of LINC01214 and miR-497-3p. The abundance of LINC01214 increased (c), while miR-497-3p decreased (d) in the NSCLC cell lines. (e) Potential target binding sites between LINC01214 and miR-497-3p. (f) Luciferase to verify the targeting relationship.

The level of miR-497-3p declined significantly in NSCLC tissues (*p* < 0.001, Figure 3(a)), showing an opposite trend to LINC01214. There was a negative correlation between miR-497-3p and LINC01214 expression in the tissue (*r* = −0.508, *p* < 0.0001, Figure 3(b)). The expression patterns of LINC01214 and miR-497-3p in four NSCLC cell lines (H-2009, PC-9, A549, and H1299) mirrored those in the tissues, with LINC01214 increasing and miR-497-3p decreasing. The most significant changes were seen in the PC-9 and A549 cell lines (*p* < 0.001, Figures 3(c) and 3(d)).

The binding condition was predicted using the lncRNASNP database (Figure 3(e)). In the WT-LINC01214 group of both NSCLC cell lines (PC-9 and A549), miR-497-3p significantly reduced the relative luciferase activity (*p* < 0.001), while the miR-497-3p inhibitor led to a significant increase (*p* < 0.001, Figure 3(f)).

### 3.3. LINC01214/miR-497-3p Axis Regulated NSCLC Cell Proliferation, Migration, and Invasion

**Figure 4:**
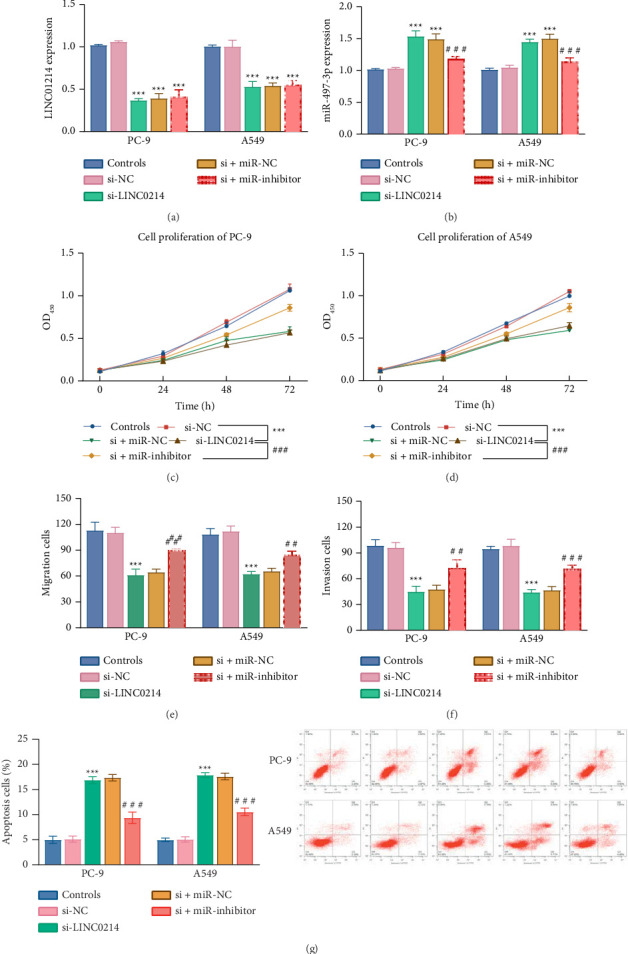
Impact of LINC01214/miR-497-3p axis on cellular activities of NSCLC cells. Silencing LINC01214 decreased the LINC01214 level (a) and increased the miR-497-3p level (b). Regulation of transfected/cotransfected miR-497-3p and LINC01214 on cell proliferation (c and d), cell migration (e), invasion (f), and apoptosis (g).

Silencing LINC01214 resulted in a significant reduction of its expression by over 50% (*p* < 0.001, Figure 4(a)). Following the silencing, no significant change in LINC01214 expression was observed after supplementation with a miR-inhibitor, with expression levels remaining lower than the control (*p* < 0.001, Figure 4(a)). In contrast, miR-497-3p expression exhibited an inverse trend under the same treatments in both PC-9 and A549 cells (*p* < 0.001, Figure 4(b)).

Proliferation rates were significantly reduced following si-LINC01214 treatment in both cell lines (*p* < 0.001, Figures 4(c) and 4(d)). Similarly, cell migration and invasion activities were markedly diminished in PC-9 and A549 cells (*p* < 0.001, Figures 4(e) and 4(f)). The apoptotic cell number significantly decreased in the si-LINC01214 treatment group (*P* < 0.001, Figure 4(g)). However, the downregulation of miR-497-3p negated the effects of LINC01214 silencing on cellular activities (*p* < 0.001, Figures 4(c), 4(d), 4(e), 4(f), and 4(g)).

### 3.4. miR-497-3p Binds to HSP90AB1 Targeting

**Figure 5:**
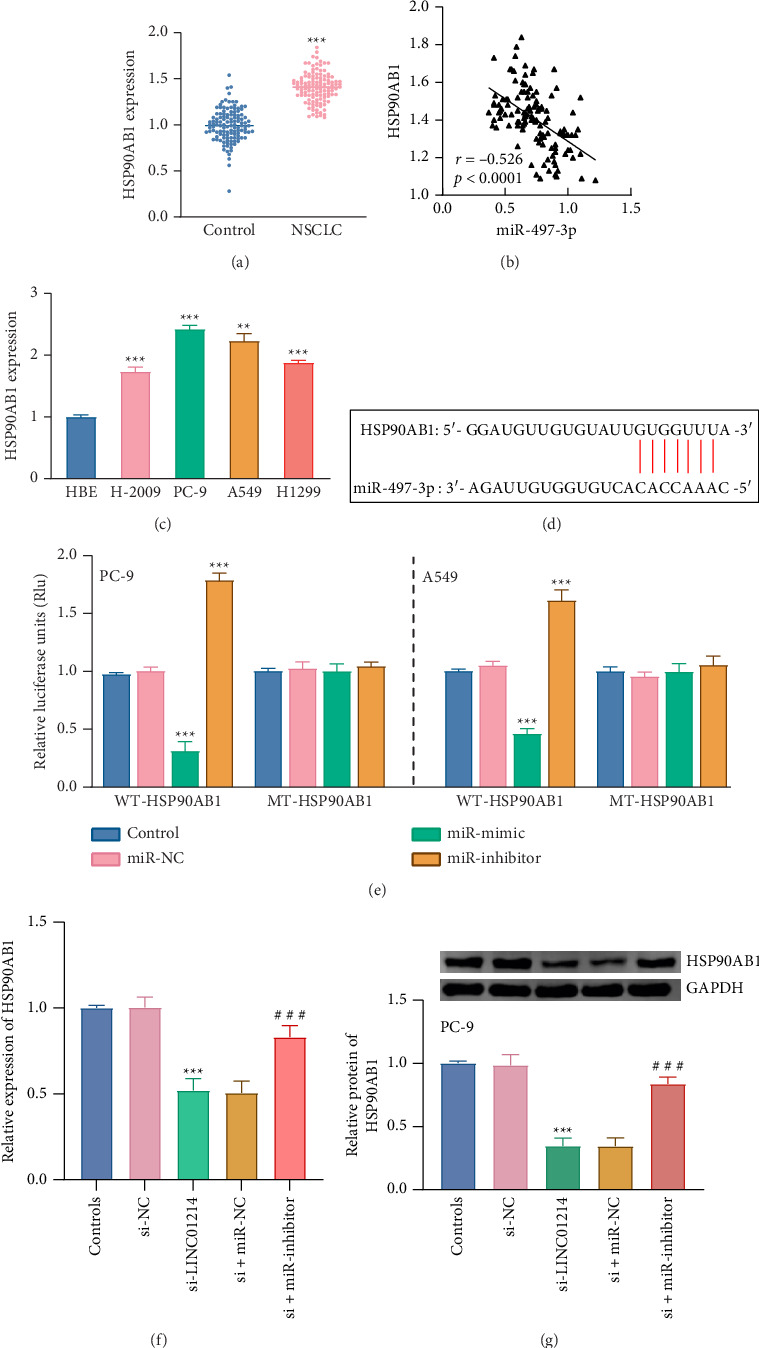
HSP90AB1 was a direct target of miR-497-3p. (a) HSP90AB1 was elevated in the NSCLC tissues. (b) Negative correlation between the level of LINC01214 and miR-497-3p. (c) The abundance of HSP90AB1 increased in the NSCLC cell lines. (d) Prediction of the target binding sites of miR-497-3p and HSP90AB1. (e) Luciferase to verify the targeting relationship. (f and g) The mRNA (f) and protein (g) levels of HSP90AB1 were examined in PC-9.

HSP90AB1 levels were significantly higher in tumor tissues (*p* < 0.001, Figure 5(a)) and were negatively linked with miR-497-3p expression (*r* = −0.526, *p* < 0.0001, Figure 5(b)). The expression of HSP90AB1 was notably upregulated in all four NSCLC cell lines, with the highest expression observed in the PC-9 and A549 cell lines (*p* < 0.001, Figure 5(c)).

The binding sites between miR-497-3p and HSP90AB1 were predicted using the lncRNASNP database (Figure 5(d)). Upregulation of miR-497-3p significantly reduced relative luciferase activity, while this trend was reversed by the miR-inhibitor in the WT-HSP90AB1 group (*p* < 0.001, Figure 5(e)). Significant differences were observed only in the WT-HSP90AB1 group, with no statistical significance in the MUT-HSP90AB1 group in both cell lines (*p* > 0.05, Figure 5(e)). In addition, the mRNA and protein levels of HSP90AB1 were examined in PC-9 cells. Treatment with si-LINC01214 significantly reduced HSP90AB1 expression at both the mRNA and protein levels (*P* < 0.001, Figures 5(f) and 5(g)). However, when miR-497-3p was simultaneously inhibited, HSP90AB1 expression was partially restored compared to si-LINC01214 treatment alone (*p* < 0.001, Figures 5(f) and 5(g)).

**Figure 6:**
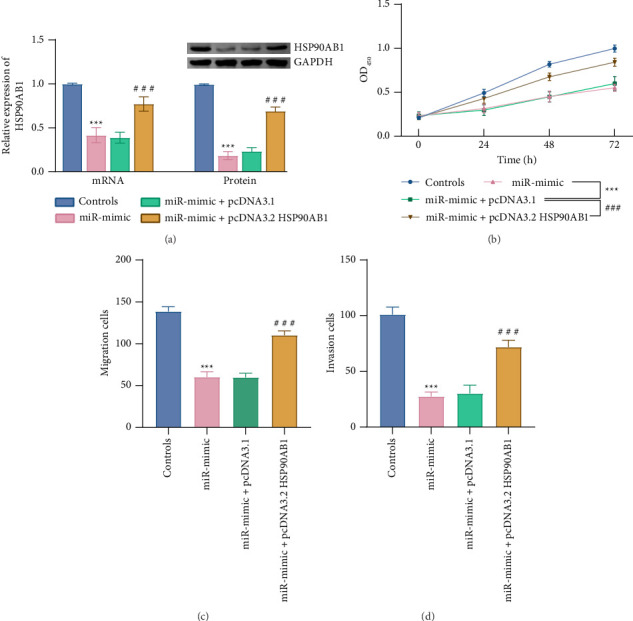
The LINC01214/miR-497-3p/HSP90AB1 axis regulated NSCLC cell activities. (a) The relative mRNA and protein levels of HSP90AB1 in PC-9. (b–d) Impact of LINC01214/miR-497-3p/HSP90AB1 axis on PC-9 cell proliferation (b), migration (c), and invasion (d).

Rescue experiments further validated the regulatory axis: upregulation of miR-497-3p partially suppressed HSP90AB1 mRNA and protein expression (*p* < 0.001, Figure 6(a)) and inhibited PC-9 cell proliferation, migration, and invasion (*p* < 0.001, Figures 6(b) and 6(c)). However, cotransfection with HSP90AB1 overexpression restored HSP90AB1 expression (*p* < 0.001, Figure 6(a)) and reversed the inhibitory effects on cell proliferation, migration, and invasion (*p* < 0.001, Figure 6(d)).

## 4. Discussion

The growth and division of cancer cells in NSCLC are slow, and the spread and metastasis of small cell carcinoma are late [20]. NSCLC is usually diagnosed at an advanced stage, which cannot be treated surgically due to local invasion or distant metastasis [21]. Therefore, the search for new effective therapeutic targets is crucial [22]. LncRNAs are closely related to the diagnosis, prognosis, and drug resistance of tumors [23]. In this study, LINC01214 was found to be significantly upregulated in malignant tissue, which was aligned with previous studies [12]. The clinical significance of LINC01214 was further investigated. Low LINC01214 levels seem to link with lower mortality. Cox regression analysis also showed that cancer grade indicators and LINC01214 were independent predictors of death in NSCLC patients. Therefore, LINC01214 might be a prognostic biomarker for NSCLC. The correlation analysis between LINC01214 and clinical indicators of NSCLC patients showed that the LINC01214 abundance was strongly associated with differentiation, lymph node metastasis, and TNM stage. The degree of tumor differentiation is an indicator of tumor malignancy [24]. Lymph node metastasis of pulmonary carcinoma is the main source of morbidity and mortality of lung cancer [25]. The TNM staging system is an important tool for evaluating the severity and prognosis of malignancy. Based on these results, it is proposed that LINC01214 is associated with tumor progression in NSCLC. All these suggest that LINC01214 is a biomarker in NSCLC prognosis.

Research into LINC01214 is not in-depth. The present finding suggested that LINC01214 might contribute to the immune pathogenesis of psoriasis by activating or inhibiting related immune cells [14]. However, the role of lncRNA in NSCLC remains unclear so far. This study found that LINC01214 was significantly upregulated in NSCLC tissues and cell lines and was associated with poor prognosis, suggesting its potential role in promoting cancer invasiveness. Cancer cell proliferation, migration, and invasion are critical biological processes that drive tumor progression and metastasis. It is reported that knocking down TTYH3 reduces the migration and invasion of A549 cells, implicating TTYH3 in NSCLC metastasis [26]. Silencing RP11-297P16.4 inhibited the proliferation and invasion of A549 and H1299 cells, highlighting its role in NSCLC progression [27]. In our study, knockdown of LINC01214 significantly suppressed the proliferation, migration, and invasion of NSCLC cell lines, further supporting its oncogenic role in tumor progression.

LncRNAs can act as sponges for miRNA, which is one of the mechanisms by which they influence the development of NSCLC [28, 29]. For example, miR-940 inhibits cancer cell proliferation by targeting FAM83F and then inhibits NSCLC progression [30]. LINC00313/miR-4429 axis is connected with the prognosis of NSCLC [31]. In the present study, silencing LINC01214 reduced the NSCLC cellular activities including proliferation, migration, and invasion. While miR-497-3p could reverse the impact of LINC01214 on NSCLC cells. We speculated that miR-497-3p acted as an endogenous competing RNA downstream of LINC01214 and was negatively regulated by LINC01214 in a mechanistic manner. Rapid growth of malignant cells is a key part of cancer progression, allowing the disease to evolve quickly and become more aggressive. This uncontrolled growth occurs in the tumor stage, both the early and metastasis [32]. Cancer cell migration and invasion facilitate tumor metastasis by crossing the basement membrane and penetrating the extracellular matrix before entering the circulatory system [33]. Reports have shown that miRNAs are involved in uncontrolled cell proliferation, replication, immortalization, apoptosis resistance, and metastasis, acting as tumor suppressors or oncogenic miRNAs [34, 35]. For instance, ELFN1-AS1/miR-497-3p regulates the ovarian cancer cells' proliferation, invasion, and migration [16]. Therefore, it is speculated that the promotion of LINC01214 on NSCLC cell proliferation and migration was mediated by miR-497-3p.

To investigate the molecular mechanism of miR-497-3p, its targeted gene was predicted by bioinformatics databases. Potential binding sites between miR-497-3p and HSP90AB1 were identified. HSP90AB1 plays an important regulatory role in tumors [36]. For example, Cortex Lycii may inhibit epithelial–mesenchymal transition (EMT) in lung cancer cells by targeting HSP90AB1 [37]. In addition, EEF1A2 interacts with HSP90AB1 to promote lung adenocarcinoma metastasis [38], and DSCC1 interacts with HSP90AB1 to enhance the proliferation and metastasis of lung adenocarcinoma cells [39]. HSP90AB1 was markedly elevated in both NSCLC tissues and cell lines, and it exhibited a negative correlation with miR-497-3p expression. These findings suggested that LINC01214 promoted the proliferation and metastasis of NSCLC cells and contributed to NSCLC development via the miR-497-3p/HSP90AB1 axis. This study has some limitations. Although this study demonstrated that LINC01214 regulates NSCLC cell proliferation, migration, invasion, and apoptosis, we did not assess EMT marker expression. While some studies suggest that EMT is linked to drug resistance, others have reported no significant association between EMT and overall survival [40, 41]. Given the ongoing debate regarding the role of EMT in NSCLC progression [42], this aspect was not explored in the current work. Future studies will aim to investigate whether LINC01214 contributes to NSCLC progression through EMT-related pathways. Although the targeting relationship between miR-497-3p and HSP90AB1 was experimentally validated in this study, additional databases could be utilized during the target prediction stage to enhance the integration of bioinformatic resources. Future studies should further incorporate and validate target interactions using a wider range of bioinformatics tools and supporting experimental data. Another limitation of this study is the heterogeneity of treatment regimens, including surgery, chemotherapy, and targeted therapy, as well as the relatively small sample size within treatment subgroups. As a result, treatment variables were not included in the Cox regression model. These factors may influence patient prognosis and should be accounted for in future studies with more comprehensive and standardized treatment data. One limitation of this study is the lack of in vivo validation to assess the effects of LINC01214 knockdown on tumor growth and metastasis. Although preliminary plans for animal experiments were considered, they could not be completed due to constraints in funding and technical capacity. Future studies will focus on conducting animal experiments to confirm and extend our in vitro findings. As this is a preliminary study, our primary focus was to assess the individual prognostic value of LINC01214 and to explore the regulatory mechanism of the LINC01214/miR-497-3p/HSP90AB1 axis in NSCLC. Future research will aim to incorporate more relevant, valuable literature and enhance the rationality and rigor of the experimental design [43].

## 5. Conclusion

In conclusion, this study established lncRNA LINC01214 as a prognostic biomarker for NSCLC patients. LINC01214 contributes to NSCLC progression by negatively regulating miR-497-3p, further promoting cell proliferation, migration, and invasion through HSP90AB1. These findings offer a theoretical foundation for the clinical development of new treatment strategies.

## Figures and Tables

**Table 1:** Relationship between LINC01214 expression levels and clinical indicators in NSCLC.

Parameters	Cases (*n* = 122)	Low LINC01214 (*n* = 59)	High LINC01214 (*n* = 63)	*p* values
Age (years)				0.713
< 60	60	28	32	
≥ 60	62	31	31	
Gender (male/female)				0.200
Male	63	34	29	
Female	59	25	34	
Smoking history				0.129
No	52	21	31	
Yes	70	38	32	
Tumor size				0.818
≤ 3 cm	69	34	35	
> 3 cm	53	25	28	
Differentiation				0.039
Well-moderate	82	45	37	
Poor	40	14	26	
Lymph node metastasis				0.023
No	83	46	37	
Yes	39	13	26	
TNM stage				0.043
I-II	80	44	36	
III	42	15	27	
Neoplasm stage				0.216
T1-T2	78	41	37	
T3-T4	44	18	26	

## Data Availability

The datasets used and/or analyzed during the current study are available from the corresponding author on reasonable request.

## Supporting Information

Supporting Table S1: STR loci of cells. This table presents the short tandem repeat (STR) profiling data for the cell lines used in this study.

Supporting Figure S1: The expression levels of LINC01214 in the lung adenocarcinoma (LUAD) and lung squamous cell carcinoma (LUSC) cohort data. This figure shows the TPM levels of the LINC01214 in LUAD and LUSC samples from the TCGA database.
