# Supplementary material for: LINC01214 Promotes Non-Small Cell Lung Cancer Through the miR-497-3p/HSP90AB1 Axis
Source: Can Respir J. 2025 Oct 30;2025:5575392. doi: 10.1155/carj/5575392 (PMC12591826; doi:10.1155/carj/5575392)
Supplement: Supporting Information — Additional supporting information can be found online in the Supporting Information section. [file 5575392.f1.docx]

Supp Table 1 STR Loci of samples

| STR Loci | PC-9 | A549 | H1299 | H2009 | HBE |
| --- | --- | --- | --- | --- | --- |
| Amelogenin | X | X,Y | X | X | X,Y |
| D5S818 | 11 | 11 | 11 | 13 | 12 |
| D13S317 | 8 | 11 | 12 | 12 | 8,11 |
| D7S820 | 10,11 | 8,11 | 10 | 9,11 | 9,10 |
| D16S539 | 9 | 11,12 | 12,13 | 12 | 9,12 |
| vWA | 17 | 14 | 16,17,18 | 14 | 16,17 |
| TH01 | 7 | 8,9.3 | 6,9.3 | 9.3 | 5,6 |
| TPOX | 11 | 8,11 | 8 | 8 | 8,10 |
| CSF1PO | 11 | 10,12 | 12 | 10,12 | 9 |
| D19S433 | 13,15.2 | 13 | 14 | 14,16,17 | 13,16 |
| D21S11 | 29,30 | 29 | 32.2 | 29 | 28,30 |
| D18S51 | 15 | 14,17 | 16 | 14 | 12 |
| D6S1043 | 13,19 |  |  |  | 13 |
| D3S1358 | 16 |  |  | 16 | 16,17 |
| Penta D | 9,13 |  |  |  | 9,12 |
| D2S441 | 11 |  |  |  | 10,11 |
| D8S1179 | 11,15 |  |  | 10,15 | 13,14 |
| Penta E | 11,15 |  |  |  | 12,16 |
| D12S391 | 18 |  |  |  | 19,24 |
| D2S1338 | 19,20 |  |  | 16,19 | 16,17 |
| FGA | 23,25 |  |  | 22 | 24,25 |

Supp Figure


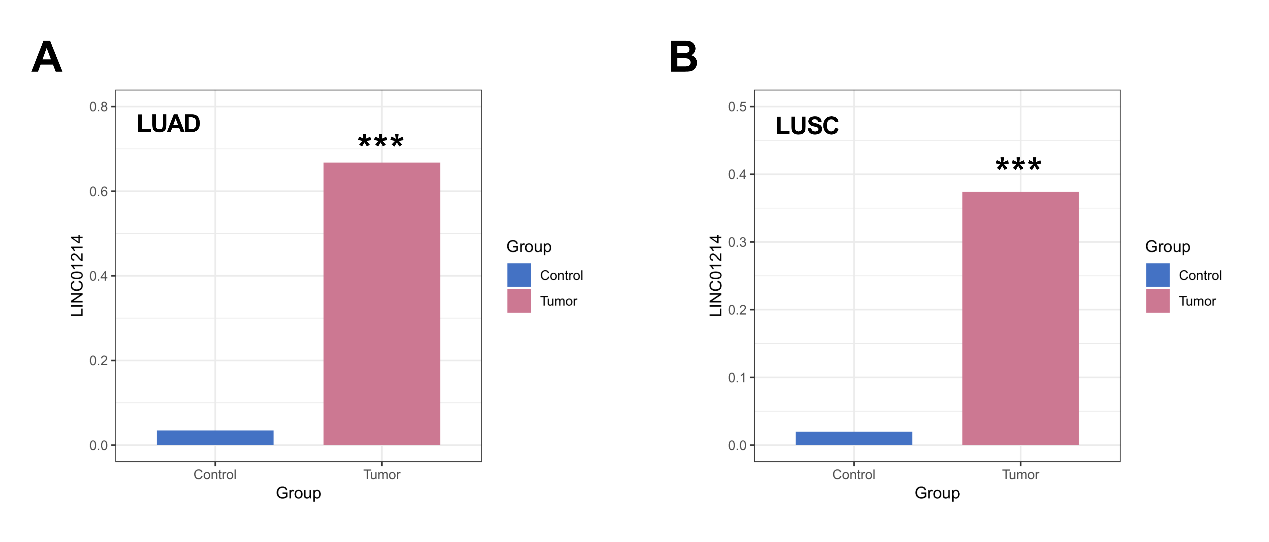


The TPM levels of LINC01214 in TCGA LUAD (A) and LUSC (B) cohort data.
